# Does the use of hormonal contraceptives induce negative metacognitions, brooding, and depressive symptoms in an adolescent community sample?

**DOI:** 10.1016/j.cpnec.2026.100386

**Published:** 2026-10-19

**Authors:** Leif Edward Ottesen Kennair, Marius Stavang, Ingrid Grønnæss, Helene Borgen Myhr, Roger Hagen, Trond Viggo Grøntvedt, Steven W. Gangestad, Mons Bendixen

**Affiliations:** a Department of Psychology, Norwegian University of Science and Technology, Trondheim, Norway; b Barne- og familiesenteret (Children and Family Center), Holmestrand Municipality, Holmestrand, Norway; c Department of Psychology, University of Oslo, Oslo, Norway; d Research Institute, Modum Bad, Vikersund, Norway; e Department of Psychology, University of New Mexico, USA

## Abstract

In western countries women are much more likely to suffer from depression than men are. Some reports have suggested that there is an increased risk of depression diagnoses in young women who start using hormonal contraception. Using an established approach to quantifying levels effective dosages of estradiol, progestins, and androgens in specific brands and types of hormonal contraception, we address a) whether hormonal contraception use in general is associated with depressive symptoms, and b) which contraceptive hormonal effects are associated with depressive symptoms as measured with the Beck Depression Inventory, brooding and negative metacognitions about rumination in a community sample of 757 young women (age 16-19). We also consider associations with age and relationship status. We detected no overall effect of hormonal contraception on depressive symptoms. We furthermore detected no effects of specific hormonal dosages on any of the three outcome measures depressive symptoms, brooding or negative metacognitions. The results are discussed in light of previous robust but limited findings of an association and the possibility that health care system contact increases likelihood of more health care services, rather than robust causal effects of hormones.

## Highlights

• In a community sample age 16-19 there is no association between HC use and depressive symptoms.
• There are no associations between specific effective dosages of HC hormones and symptoms of depression, negative metacognitions, or brooding.
• This suggests that there may not be a direct link between HC use and depression, and one needs to consider other pathways to explain robust but limited findings of associations between HC use and MDD.

## 1 Introduction

Depression is rated as the leading cause of disability among adults worldwide, and affects more women than men [1]. Depressive symptoms among adolescent and young adult women are further on the rise, documented in many nations, albeit most notably Western countries [2]. There is a high prevalence of depressive symptoms in adolescence [3], [4], [5], and particularly for girls [6]. This sex difference appears in adolescence [7], [8]. There is scarce research on how and why the sex difference in depressive symptoms increases during adolescence. One hypothesis is that these symptoms are fostered partly by female pubertal hormones and the use of hormonal contraceptive (HC) [9], [10]. Despite some very convincing evidence from large register studies (e.g., Ref. [11]), this hypothesis is controversial [12]. The current study considers HC use and how specific HC hormones are associated with depressive symptoms and with factors known to foster and maintain depressive symptoms such as metacognitions and brooding [6], [13].

Multiple factors have been suggested to contribute to the sex difference in depressive symptoms: gender socialization [14]; differences in frequency of negative life events; differences in rate of unemployment [15]; heritable predispositions for depression [16] and burden of genetic risk [17]; differences in levels of rumination [6], [18], [19]; and differences in negative metacognitions [6]. The sex gap is particularly prominent after girls' menarche [20], from 12 to 15 years, when adolescent women's levels of estrogens and progesterone increase [21].

Synthetic forms of estrogenic and progestogenic hormones, are used in hormonal contraception, and it is plausible that hormonal contraception could affect levels of depressive symptoms in adolescent women. In a national prospective study of more than one million Danish women, Skovlund et al. [11] reported that hormonal contraception was robustly associated with increases in diagnosed depression and the use of anti-depressants. More recently, Anderl et al. [22] also report a small increase in MDD diagnosis after HC use; however, they did not let prior MDD be a competing predictor (and this was their strongest predictor). By contrast, others have found no meaningful association between HC usage and symptoms of depression, while some have even found a relief of depressive symptoms while on HC [23], [24], and that discontinuation of HC is associated with an increase in depression [25].

Given these conflicting findings, studies have also attempted to assess whether the claimed relation between HC and symptoms of depression is based on chemical make-up of the contraceptives. This is further based on the assumed role estrogens and progesterone might play in the depression gap as girls and boys enter adolescence [11]. Estradiol (the most potent form of natural estrogens) may affect depression through its effects on the levels of neurotransmitters in the brain, particularly serotonin, dopamine, and norepinephrine [26]. Progesterone receptors are widely distributed throughout the brain in cortical and subcortical regions, and are particularly dense in regions of the hypothalamus, a structure that is believed to be particularly relevant in emotion processing [27]. Progesterone may affect depressive and anxiety symptoms, at least in part, through allopregnanolone pathways. Allopregnanolone, a metabolite of progesterone, is known to potently upregulate transmission at GABA-A receptor sites. GABA is an inhibitory neurotransmitter; deficits in GABAergic transmission have been observed to promote depressive and anxiety symptoms [28]. One might expect that increases in progesterone and, accordingly, allopregnanolone levels will suppress depressive symptoms, mediated through positive effects on GABA transmission. However, evidence is mixed, leading authors to suggest that, under some conditions, allopregnanolone paradoxically promotes negative mood (for contrasting perspectives, see, e.g., Ref. [29], [30]). Bäckström et al. [31] proposed that allopregnanolone levels have an inverted-U relationship with mood symptoms, with levels characteristic of a natural luteal phase associated with higher expression of depressive symptoms, and either lower or higher levels of allopregnanolone associated with reduced depression. Other scholars suggest that effects are moderated by individual differences, with some women being particularly sensitive to the negative impacts of progesterone (e.g., Ref. [32]).

Contraceptives differ in the chemical structure of their constituents. Some hormonal contraceptives are based on a mixture of progestin and estradiol, while others are purely based on progestin. In addition, forms of progestin vary. First through third generation progestins (e.g., levonogestrel, desogestrel) are androgens, which have androgenic as well as progestogenic effects. A fourth generation progestin, drospirenone, has modest anti-androgenic impacts. Studies examining the effects of contraceptives with specific progestins, or the impacts of switching from a contraceptive with one progestin to another one, have yielded mixed results ([32], [33], for reviews). Some studies suggest that progestins with greater androgenic effects promote depressive symptoms, relative to progestins with anti-androgenic effects (e.g., Ref. [34]). Despite some work finding increased levels of depressive affect in women using progestin-only contraceptives [35], the literature as a whole finds no clear-cut association between progestin-only formulations and negative affect.

Studies have also investigated whether the association between depression and hormonal contraception is dependent upon the route of administration. No clear association between route of administration of HC and depression has been established (for reviews, see Ref. [32], [36]). The outcome measures of most of these studies were typically depression diagnosis or usage of anti-depressants. We aim to investigate whether routes of administration might be more clearly associated with levels of symptoms of depression, rather than presence or not of diagnosis. When HCs are delivered via a patch, an injection, an implant, or an IUD, estradiol and progestin levels delivered are typically lower, as a sizable proportion of hormones ingested in a pill are quickly taken out of the bloodstream as a result of first-pass metabolism in the liver [37].

From an evolutionary perspective, the field is not well enough informed about the full range of functional effects of estrogen and progesterone to yield clear predictions about specific dysfunctional or unintended outcomes. There has been a long-standing interest in understanding the hormonal underpinnings of premenstrual symptoms that some women experience. But progesterone levels do not peak during the premenstrual phase. And a meta-analysis yielded no robust patterns of hormonal influences [38]. However, administering artificial hormones to a population might influence development, mood, and sexual behavior, and may result in population level dysfunction. Despite mounting interest and some caution about negative effects [39], there remains too little research on how HC influences women's psychology.

While, in this current sample, as in western countries in general, we previously found that the most severe symptom scores are much higher for girls than boys, boys’ symptom scores increase across puberty too [6]. As we reported in the previous study, girls also had higher brooding scores and negative metacognitions. These are statistically associated factors with increased depressive symptoms [6].

### 1.1 The current study

The main aim of the current study is to examine how depressive symptoms along with factors known to predict depressive symptoms such as negative metacognitions and brooding are influenced by hormonal contraceptive use in a sample of Norwegian female Upper Secondary School students.

First, based upon findings that suggest that there is an (causal) association between HC use and depression, we sought to replicate the effect of HC on depressive symptoms and extend the analysis to negative metacognitions and brooding.

Thus, we predict that overall hormonal contraception usage will be associated with an increase in symptoms of depression.

Second, using hormone levels and potencies across HC brands [40], we investigated how, within HC users, particular contraceptive hormonal constituents (specific components with estragenic effects, progestogenic effects, androgenic effects) covary with depressive symptoms, brooding or negative metacognitions in our sample. Separately, we examined the impact of routes of administration.

Our study employs the Beck Depression Inventory as an outcome measure, in conjunction with measures of Negative Metacognitions and Brooding, which were established as highly associated with depressive symptoms in Pedersen et al [6], to provide a more finely grained, and quantitatively variable, assessment of symptoms of depression rather than relatively coarse present/absent assessment of diagnosis available in register studies. We expect that these measures are potentially more sensitive to detecting any associations between HC and depression compared to a coarser register diagnosis status. However, we also examined associations of HC use with treatment for mental health issues and depression.

## 2 Methods

### 2.1 Participants

Students enrolled in Upper Secondary schools in the Trøndelag and Møre and Romsdal counties were invited to respond to a questionnaire on depressive symptoms, rumination, metacognitions, and hormonal contraception use in 2019. The sample covered both vocational and college-preparatory upper secondary schools from both rural and urban (i.e., schools from Norway's third largest city) areas. Response rate was not recorded. Of the 1418 responses, only students aged 21 or younger, and who identified as man or woman were included. We excluded students who did not fill in the final part of the questionnaire on depressive symptoms, and following screening procedures also students with monotonous responding. This left us with 1198 (87.2%) responses eligible for analyses (for more details, see Ref. [6]). For the present study, data from women only were analyzed (*n* = 757, aged between 16 and 20, *M*_age_ = 18.1, SD = 1.1).

### 2.2 Procedure

After inviting the principals, school nurses, counsellors, and psychology teachers at the upper secondary schools in Trøndelag and Møre and Romsdal counties, the principals at the attending schools were contacted to distribute a link to the online questionnaire along with information about the study to the students. Students in vocational studies who were in practice were also invited by their school principal. There were no external incentives offered for participation, and all responses remained anonymous (IP-addresses were removed from the file before analysis). The Regional Committees for Medical and Health Research Ethics approved the study and design (ref. no. 2017/2062).

### 2.3 Measurements

To assess depressive symptoms, we administered the full 21-item Beck Depression Inventory (BDI) [41]. The BDI covers cognitions, feelings and behaviors typically associated with depression. For each item, participants gave their responses on a scale ranging from 0 to 3, with higher values reflecting more intense or severe symptoms (e.g., guilt, dissatisfaction, sadness, stamina, and sleep). The individual items were summated to form a scale. The BDI is validated for younger, non-clinical populations. Internal consistency for this sample was very good (*α* = .92).

To assess HC use, we posed several questions. The participants first responded “Yes” or “No” to the question: Do you currently use hormonal contraception, such as pills, hormonal spiral, minipill, rod, injection, or patch? If they responded “Yes”, they were then asked to specify what kind (pills, rod, patch, etc.) and the brand of HC using pre-defined pull-down menus. Next, they were asked for how long they had used the product, whether they had changed product within the past 2 years, and, if “Yes”, what brand they changed from and when (how long ago).

The synthetic estrogen used in HCs is uniformly ethyl estradiol. The potency of a dose for ethyl estradiol then, is captured simply by the dose amount. By contrast, progestins take a number of different forms (e.g., particularly common progestins include desogestrel, levonorgestrel, and etonogestrel, drospirenone). Progestins have different potencies—that is, they differ with respect to the dosage that has physiological effects (e.g., the dosage that is sufficient to reliably prevent pregnancy, e.g., the effective dose of drospirenone is much greater than the effective dose of the other three progestins listed above). As well, while some progestins have androgenic effects (including desogestrel, levonorgestrel, and etonogestrel), others have anti-androgenic effects (e.g., drospirenone). Previously, Grøntvedt et al. [40] used the literature on dosages and potencies to place the progestogenic effects of each dosage/potency of specific progestins on a common scale. They also developed a measure of the androgenic effects of progestins. See Grøntvedt et al. [40], Table 2, for details. For ease of communication, we refer to the estimated estrogenic, progestogenic, and androgenic effects of the constituents of HCs as effective dosages of estrogens, progestins, and androgens.

The brooding aspect of rumination, reflecting negative or moody connotations, was measured using a 5-item short version of Ruminative Response Scale [42]. We asked each participant to consider the last times they had felt sad or had a problem and to report how frequently they had thoughts such as: “Why do I have problems other people don't have?”, “What have I done to deserve this”. Responses ranged from 1 (*Almost never*) to 4 (*Almost always*). Internal consistency was good (*α* = .82). Scores were averaged to form a brooding scale.

We administered the 13-item version of the Negative Beliefs about Rumination Scale (NBRS; [43]) to assess negative metacognitions such as ‘People will reject me if I ruminate’ and ‘Ruminating about my problems is uncontrollable.’ Scores ranged from 1 (*Disagree*) to 4 (*Totally agree*). Internal consistency was very good (*α* = .90). Scores were averaged to form a negative metacognitions scale.

In addition to the above we asked about their relationship status (32% reported being partnered, 54% reported being single, 14% in some kind of situationship). We also asked if they currently received any treatment for mental disorders (Yes = 20.7%), if they currently received treatment for depression specifically (Yes = 11.6%), and if they currently used antidepressants prescribed by their doctor (Yes = 6%). Of the 82 who currently received treatment for depression, 26 also used antidepressants.

### 2.4 Analyses

All analyses were performed using StataNow/MP 18.5 for Mac [44]. For group comparisons, we report Hedges *g* as an indication of the size of the difference. For bivariate associations, we report Pearsons's *r*. For predicting negative metacognitions, brooding, and depressive symptoms from hormonal variables (with interactions), we performed hierarchical regression analysis. Core assumptions of linearity, homoscedasticity, normality of residuals, and multicollinearity were checked throughout and reported when not met. Robust estimates of standard errors were applied that resolve some of issues following heteroscedisticity and outliers. In Model 1, we regressed depressive symptoms on effective dosage of progestin, estradiol, androgens, and delivery method. In Model 2, we added to the above the interactions between delivery method (oral or not) and each of the hormone dosages. Post hoc power analyses of the regression analyses were performed using G*Power 3.1.

## 3 Results

### 3.1 Descriptives and associations between depression-related outcomes

Hormonal contraceptive (HC) use was common in this sample (62%, *n* = 467). Brand type was identified for 439 women (Table 1). These were coded for level of estradiol, androgens, and progestin for analysis of effect on depressive symptoms. The majority of HC users reported oral delivery (57%), followed by implant (32%) and intrauterine (7%). Mean age at first menarche (*n* = 687) was 13.0 years (SD = 1.5). Mean age for onset HC use (*n* = 440) was 16.3 years (*SD* = 1.5), and mean age for duration of HC use was 2.0 years (*SD* = 1.6). Forty percent reported having swapped HC brand and/or type during the past 2 years.

**Table 1:** Number of participants who report using specific oral or non-oral hormonal contraceptives.

HC type/brand	Oral	Non-oral
Loette	78	
Almina	49	
Orlacon	37	
Yasmin	23	
Cerazette	21	
Microgynom	21	
Mercilon	11	
Desogestrel/Orifarm	6	
Qlaira	1	
Marvelon	1	
Diane	1	

Rod		147
Mirena		26
Jaydess		6
Ring		4
Injection		4
Patch		4

Total	*n* = 249	*n* = 190

**Table 2:** Pairwise associations (Pearson's *r's*) between demographics, HC use, antidepressant, treatment, and the three outcome variables (*n* = 701, listwise).

	1	2	3	4	5	6	7	8
1. Age	--							
2. Partnered (0,1)	.07	--						
3. HC (0,1)	.21**	.31**	--					
4. Antidepressants (0,1)	.05	.04	.04	--				
5. Treatment w/depression (0,1)	.04	.06	.08*	.39**	--			
6. Treatment wo/depression (0,1)	−.02	.06	.05	−.04	−.11	--		
7. Negative metacognitions	−.02	.12**	.04	.21**	.33**	−.01	--	
8. Brooding	−.04	.10**	.02	.16**	.27**	.04	.67**	--
9. BDI	−.03	.06	.00	.22**	.39**	.01	.76**	.66**

*Note*. *p < .05, **p < .01. w = with, wo = without. HC = Hormonal contraceptives, BDI = Beck Depression Inventory. Dichotomous predictors are marked as (0,1).

The mean level of symptoms of depression was quite high in this sample (*M* = 15.6, *SD* = 11.3; this mean score falls within a range typically taken to reflect mild depression). Antidepressant users (*M* = 25.6) scored markedly higher than non-users (*M* = 14.9) on the BDI scale, *t*(748) = 6.37, *p* < .01, Hedges *g* = .98. Those in treatment for depression (*M* = 28.1) scored markedly higher than students not receiving any such treatment (*M* = 13.8) on the BDI scale, *t*(731) = 12.00, *p* < .01, Hedges *g* = 1.38. Further, age and being partnered were positively associated with hormonal contraceptive (HC) use. Using antidepressants and receiving treatment for depression both correlated positively with negative metacognition, brooding, and depressive symptoms (see Table 1). Treatment for conditions other than depression was not associated with these outcome variables. As expected, strong associations were reported between negative metacognition, brooding, and depressive symptoms.

### 3.2 Associations with HC use

Focused analyses examining bivariate associations of depression and related outcomes (Pearson *r*) are reported in Table 2, column 3. The association of HC and depression (BDI) was .00. Associations with related measures of brooding and negative metacognitions were similarly small and non-significant, .02 and .04. We did detect an association between HC use and treatment for depression. A follow-up analysis found that the odds of being treated for depression were 78% higher for HC users (13.7%) compared to non-users (8.2%), *z* = 2.24, *p* = .025, OR = 1.78.

In Table 3 we present focused analyses examining how strongly hormonal dosage, method of delivery (oral or not), age at menarche, onset, and duration of HC use, as well as having swapped HC brand within the past 2 years, were associated with depression-related variables. As evident from the table, none of the predictors showed any significant association with negative metacognition, brooding, or depressive symptoms. Pearson *r*s with BDI were, once again, small, .01 to .03 (see also Fig. 1, Fig. 2, Fig. 3).

**Table 3:** Pairwise Associations (Pearson's *r's*) Between the Predictors and the three Outcome variables Negative Metacognition (NBRS), Brooding, and Depressive Symptoms (BDI).

	NBRS	Brooding	BDI
Progestin	−.04	.02	.01
Estradiol	−.02	.04	.01
Androgens	.02	.04	.03
Oral (0,1)	.00	.01	.03
Age at menarche	−.03	−.02	−.05
Onset HC use	−.02	−.06	−.04
Duration HC use	−.03	.00	−.01
Swapped HC	−.02	−.02	.00

Mean (SD)	1.66 (.61)	2.39 (.76)	15.58 (11.26)
Skew	1.06	.10	.76

*Note*. *n*'s 428–458 (except for Age at menarche, *n*'s = 685-687). No associations are significant at *p* < .05.

**Fig. 1:**
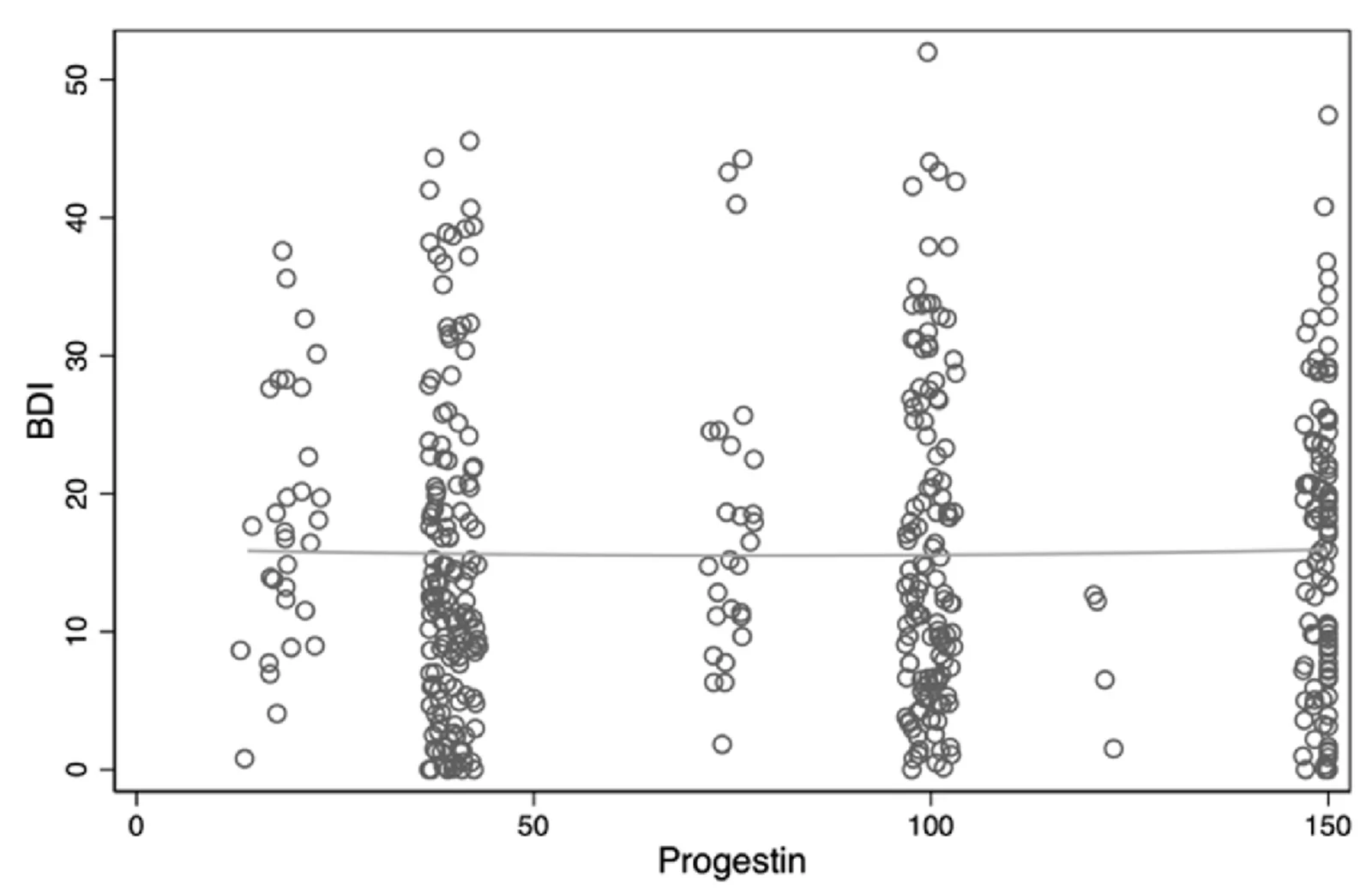
The Progestin – BDI association with quadratic fitted line.

**Fig. 2:**
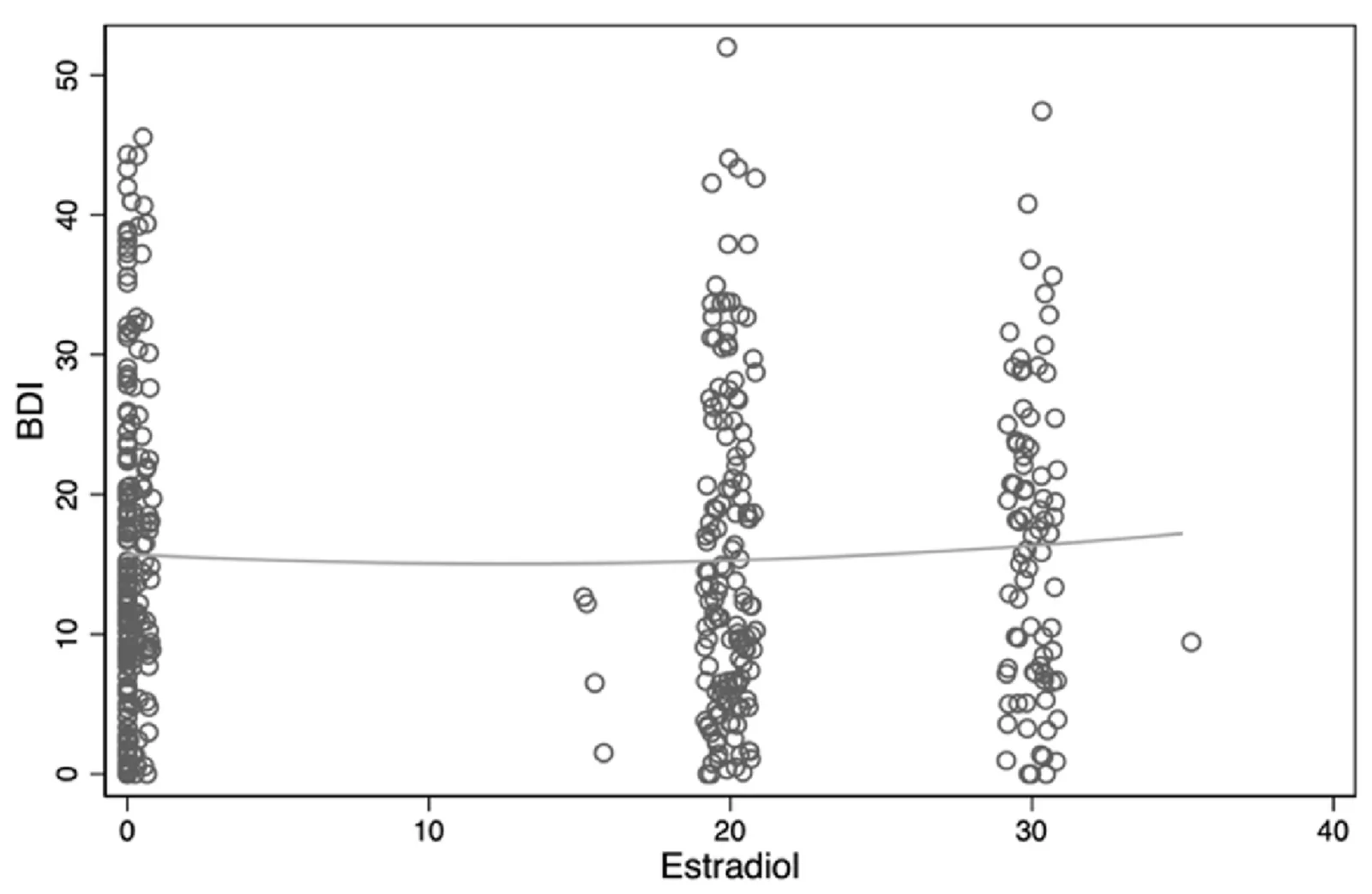
The Estradiol – BDI association with quadratic fitted line.

**Fig. 3:**
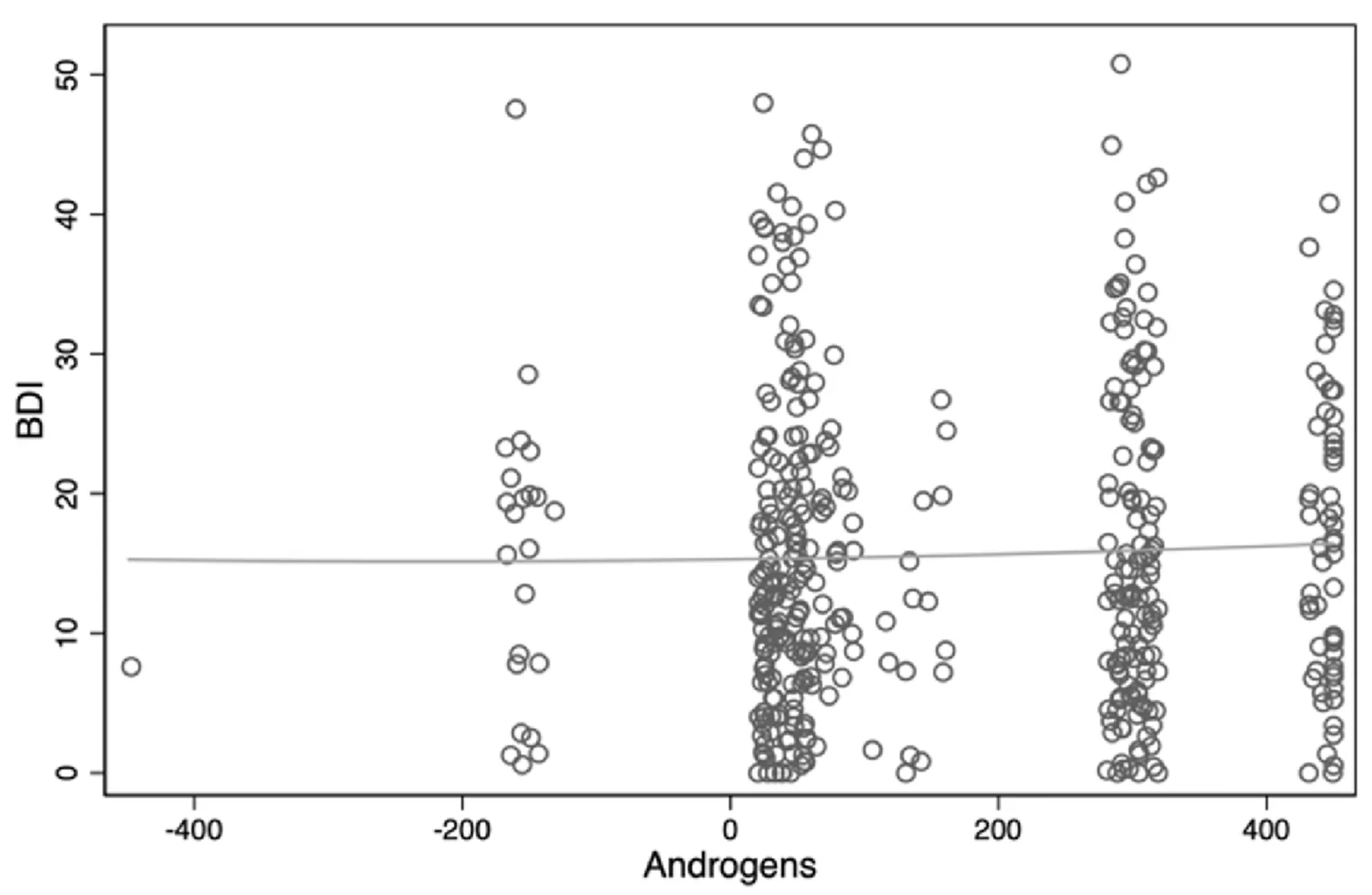
The Androgens – BDI association with quadratic fitted line.

For further illustration, we present the bi-variate association between Progestin, Estradiol, and Androgens on the one hand and BDI on the other.

### 3.3 Regression analyses

Despite the lack of bivariate associations between hormonal effective dosages and the three depression-related outcomes, we performed multivariate regression analyses on each of the outcome variables, treating effective dosages as predictors, while also examining interactions with delivery method (oral or not). Estradiol and progestin dosages were highly correlated (*r* = .95), partly because there a number of hormonal contraceptives contain no estrogen but also contain relatively weak dosages of progestins. Those not containing estradiol tend to be ones that are delivered by methods other than oral. Controlling for delivery method, the estradiol – progestin correlation dropped to *r* = .77, which is still potentially problematic with respect to multicollinearity (non-oral, *n* = 182: *r* = .84, oral, *n* = 248: *r* = .85). Androgenic effects correlated *r* = .57 with progestogenic effects, and *r* = .64 with estradiol dosage, respectively (non-oral: *r* = .48 and *r* = .86, oral: *r* = .09 and *r* = .27).

In Table 4, we present regression analyses of hormonal dosage and delivery method (Model 1). For none of the outcomes were the overall models significant (see Table note). Furthermore, none of the predictors within each model significantly predicted metacognition, brooding, or depressive symptoms. The negative association between progestogenic effects and negative metacognitions approached but did not reach statistical significance (*p* = .06). No comparable effects of progestogenic levels were observed for brooding or the BDI.

**Table 4:** Predictors of negative metacognition (NBRS), brooding, and depressive symptoms (BDI) in regression analyses: Model 1.

Effects	NBRS				Brooding				BDI			
	*B*	*SE*	*t*	*p*	*B*	*SE*	*t*	*p*	*B*	*SE*	*t*	*p*
Progestin	−.004	.002	−1.90	.06	−.004	.003	−1.20	.23	−.034	.040	−.84	.40
Estradiol	.009	.008	1.09	.28	.014	.010	1.45	.15	.028	.144	.20	.84
Androgens	.000	.000	.40	.69	.000	.000	.20	.84	.001	.004	.35	.73
Oral (0,1)	.122	.120	1.02	.31	−.001	.152	−.01	.99	2.569	2.164	1.19	.24

*Note.* Omnibus effects of predictors on outcomes.

NBRS Model: *F*(4, 425) = 1.19, *ns., R***^2^** = *.0092*; Brooding Model: *F*(4, 425) = .78, *ns., R***^2^** = .0064; BDI Model: *F*(4, 423) = .53, *ns*., *R*^2^ = *.0047*.

In Model 2, we added standardized interaction terms to examine if delivery method (oral or not) moderated the effects on hormone effective dosages on depression-related outcomes. They did not.

Analysis on assumptions identified problems with multicollinearity, and non-normal distribution of residuals. Hence, we performed all regression analyses with robust estimates of standard errors. Still, some caution needs to be taken regarding these findings. Post hoc analyses suggest very low statistical power for these findings (.313 for NBRS, .223 for Brooding, and .171 for BDI).

Additional regression analyses on the effect of age at menarche, onset HC use, duration HC use, and having swapped (no/yes) hormonal contraceptive brand the past two years, all produced null findings for each of the three outcomes (see Table 5 for details of BDI).

**Table 5:** The Effect of Age at Menarche, Onset HC use, Duration HC use, and Having Swapped (no/yes) Hormonal Contraceptive Brand on Depressive Symptoms (BDI).

Effects	*B*	*SE*	*t*	*p*
Age at menarche	−.264	.474	−.56	.58
Onset HC use	−.759	.494	−1.54	.13
Duration HC use	−.841	.535	−1.57	.12
Swapped HC	.300	1.10	.27	.79

*Note.* BDI Model: *F*(4, 399) = .79.

## 4 Discussion

A large Danish study found a robust link between upstart of HC and subsequent first diagnosed depressive episode [11]. We sought to replicate this effect and, furthermore, examine how the estrogenic, progestogenic, and androgenic effects of hormonal constituents of different HC brands (Grøntvedt et al. [40] relate to depressive symptoms and maintaining factors (negative metacognitions and brooding; Pedersen et al., 2021). Overall, we found no discernible effects of HC on these outcomes. Neither did we find associations of effective hormonal dosages with any of the depression-relevant measures, whether assessed through bivariate correlations or multiple regression. We did find a link between HC and treatment for depression.

Skovlund et al. [11] had a very sizable sample size, roughly a third of a million adolescents. They hence had considerable power to detect very small effects. Naturally, the current study cannot rule out very small effects. The study did have reasonable power to detect fairly small effects, however. A key difference between Skovlund et al. is that their dichotomous outcome variables had low base rates: for adolescents, cumulative antidepressant use and diagnosis of depression across years of roughly a few percent (exact values not able to be calculated from reported data); hence, the vast majority of their sample was assessed as having no treatment and no diagnosis. By contrast, the current study used, as a primary outcome measure, continuously varying depression symptomology assessed with a widely used and validated measure. Mean scores reflected mild depression. The current study, then, assessed variation not assessed by Skovlund et al. Put otherwise, within the vast subsets of women never formally diagnosed with or treated for depression with antidepressants, there surely is meaningful variation in depressive symptomology, which the current study assessed, but Skovlund et al.’s study did not.

A novel aspect of the current research is our examination of associations of effective dosages of hormonal components of HC with depressive associations. All associations were observed to be not only non-significant but quite small (e.g., all Pearson *r*'s with depression symptomology .01 to .03).

Recent reviews suggest that the link between depression and HC is less robust than the Danish numbers might suggest [27], [45]. There are several possible explanations of this lack of general effect. First, perhaps depressive symptoms are primarily a progestin effect [36], consistent with Skovlund et al.’s [11] finding a slightly higher rate ratio for women taking progestin-only HC compared to HC use overall. Yet, in our data, we found no significant effect of progestogenic properties and, indeed, descriptively the effect runs in a negative direction. Second, depression is a recurrent disorder, which may conflate issues—one is measuring effects of recurrent health care contact in addition to increased vulnerability to MDD. Considering Anderl et al. (2022) more closely, prior depression is a major predictor of later depression; however, they did not consider the minimal effect that HC would have had on top of prior MDD episode, although they could have. Third, primarily younger women are at risk. That might also be a conflation effect, as depressive symptoms increase across puberty [6]. However, the current sample is among the youngest samples considered and their levels of depression are stable across age groups, and we neither find any effects here. If the explanation is that those who experience averse emotional symptoms change the type of HC there should still be some low effect of specific hormones in this young sample. Primarily the link between HC and depression may therefore be a case of premorbid individual vulnerabilities, and we need to consider these in greater detail. Thus, it is maybe not a general, overall effect of HC, but rather an effect of other factors.

There is a way, however, in which our findings do not directly conflict with those of Skovlund et al. [11]. Those authors specifically examined effects of HC on first treatment (with antidepressants) for or diagnosis of depression, where formal diagnosis by a health care profession is likely to be linked to treatment. We too found that young women taking HCs were more likely to be in treatment for depression, with an estimated odds ratio of approximately 1.8 (78% greater risk). Interestingly, this figure matches very closely with the risk ratios for oral contraceptive use reported for adolescents by Skovlund et al.: 1.8 for first treatment with antidepressants, and 1.7 for first diagnosis of depression. In this sense, our findings replicate those of Skovlund et al. (though, we note, our data does not find a significant association between HC use and antidepressant use per se). At the same time, whereas Skovlund et al. did not have available to them independent measures of depressive symptoms or psychological features (e.g., brooding) associated with depression, our study did. And there, we found no evidence for associations with HC use.

This pattern of findings is consistent with a causal scenario in which HC use does not directly affect depressive symptomology yet, in some fashion, contact with the mental health care system comes to be associated with HC use. Indeed, given our findings, it seems that HC use comes to be associated with contact with the health care system in a way broader than diagnosis for depression or mood disorders specifically. One possibility that should be entertained is that contact with the health care system increases likelihood of HC. Relatedly, contact with health care providers to obtain a prescription of HC use could, in principle, increase the likelihood of a professional becoming aware of mental health issues. More generally, could much of what we observe in associations be simply explained by the fact that contact with the health care system increases a multiple-treatments risk? There may be supply-induced demand effects or cascade effects, but the explanation is perhaps that this is merely a result of systemic follow-up recommendations and basic diagnostic practice within mental health care. Many health care professionals are trained to screen depressive symptoms, both in schools, general practice and within mental health care services.

We do not mean to imply that the association between HC use and treatment is the outcome of overdiagnosis or overtreatment. Rather, we suggest that the probability of ascertainment (diagnosis) of depression as well as treatment for depression, appropriate or inappropriate, increases because of contact with health care services, even if the primary reason for seeking those services was obtaining a prescription for HC. It is possible that women who have not sought HC services remain underdiagnosed for depression. That said, increased contact with healthcare services *could* potentially lead to overdiagnosis and overtreatment. The way a healthcare system is designed and funded can influence the amount of care provided. For example, in fee-for-service systems, providers have a financial incentive to deliver more services—how strong this effect is in a welfare state health care system (such as in Norway and Denmark) is less clear. But there may also be clinical guidelines and mandatory symptom check lists that are in place because one fears that the patients are at risk of becoming depressed from hormonal interventions or just as a general follow up of young female patients. Either way, this may result in cross-selling effects.

The outcomes that the large Danish study examined were depression diagnosis and treatment for diagnosis. The current results raise the question: Might the associations between HC use and these outcomes reported in that study similarly be driven by contact with health care professionals, resulting in greater co-occurrence of diagnosis and treatment? If so, those results may not reflect a true association between HC use and depressive symptoms per se. Also, as indirect support of this, negative effects are maybe neither as important reason for discontinuation of HC as one has believed [46]. And, increasing depression amongst young women is probably not primarily explained by HC.

For reasons raised by an ethics review board, we could not consider a range of specific sexual and romantic behaviors in conjunction with the HC data in this community sample of adolescents. Developmental, emotional, relational, and sexual aspects of adolescents’ lives may influence their emotional well-being and states, which we wished to consider when designing this study, but were not able to examine in the current investigation. HC is associated with increased sexual activity and participation in romantic relationships but, consequently, it is also associated with increased relational conflict, emotional turmoil and other factors that also may drive depressive symptoms. Future studies should consider these unexplored factors in greater detail.

There was no significant association between age and depressive scores in girls, though a positive pattern was found for the adolescent males in the full sample [6]. This finding implies that pubertal girls reach an increased level of depression before age 16, the youngest age in this sample.

## 5 Strengths and limitations

This is a large community sample, with a large number of HC users and good variance on depressive symptoms measured with the clinically relevant full BDI. Nonetheless, power to detect very small effects (say, *r* = .05) is weak; naturally, very small effects could go undetected. Nevertheless, if the effects are so low that the associations between hormonal level measures and outcome measures are that low, the clinical significance is modest.

We asked about current use of HC. It is possible that some of the participants in the non-HC user group may have used HC in the past, even recently. A subset could have discontinued use due to depressive reactions to use. This could possibly reduce the difference between the non-HC users and the current HC users. We also don't know how large proportion of the sample that may have stopped their HC use prior to the study. However, the hormones and their effects likely dissipate shortly after quitting and they can get pregnant right after having stopped. Still, there may be months before their regular period is back. Given this, however, it is unlikely that prior HC users, even most recent ones, are still under the influence of hormones from HC. Naturally, those who quit HC use do not influence analyses conducted within the subset of users (examining effects of hormonal components).

We note that information on past treatment and/or antidepressant use was not collected in this study. We only asked about current treatment and use of antidepressants. Hence, the participants’ history of medical and psychological treatment remains unknown.

## 6 Conclusions

Why are young adolescent women increasingly reporting depressive symptoms? We have previously established that depressive symptoms are predicted by negative metacognitions and brooding in an adolescent community sample [6]. Based on the convincing evidence that HC use predicts depressive diagnosis [11] we employed the analytical approach of [40] to examine what specific hormones might influence depressive symptoms, brooding and negative metacognitions.

The current sample has a high percentage of HC users, and elevated levels of depressive symptoms, brooding and negative metacognitions, typical of adolescent and young adult women [6]. Contrary to expectations, we found no clear effects of HC on any of the planned outcome variables. We did find an association between HC and mental health treatment for depression. Whether this outcome is the odd one out or whether the multiple treatments model is a possible explanation cannot be concluded at this point. Further research into how HC, depression and sexual, relational, and emotional maturation is warranted. In conclusion, at a superficial level we seem to find what many have found diagnosis, health care and HC are associated; but at a more detailed mechanism level this does not seem to be because of a robust association between specific levels of hormones in HC and MDD symptoms or processes.

## CRediT authorship contribution statement

**Leif Edward Ottesen Kennair:** Writing – review & editing, Writing – original draft, Supervision, Project administration, Methodology, Investigation, Conceptualization. **Marius Stavang:** Writing – review & editing, Writing – original draft. **Ingrid Grønnæss:** Writing – review & editing, Methodology, Investigation, Conceptualization. **Helene Borgen Myhr:** Writing – review & editing, Methodology, Investigation, Conceptualization. **Roger Hagen:** Writing – review & editing, Supervision, Project administration. **Trond Viggo Grøntvedt:** Writing – review & editing, Methodology. **Steven W. Gangestad:** Writing – review & editing, Writing – original draft, Methodology, Formal analysis. **Mons Bendixen:** Writing – review & editing, Writing – original draft, Supervision, Project administration, Formal analysis, Data curation.

## Availability of data and materials

The dataset analyzed during the current study is available from the corresponding author on reasonable request.

## Declaration of competing interest

The authors declare that they have no known competing financial interests or personal relationships that could have appeared to influence the work reported in this paper.

## References

[1] WHO (2018).

[2] Shorey S., Ng E.D., Wong C.H.J. (2022). Global prevalence of depression and elevated depressive symptoms among adolescents: a systematic review and meta-analysis. Br. J. Clin. Psychol..

[3] Bhatia S.K., Bhatia S.C. (2007). Childhood and adolescent depression. Am. Fam. Physician.

[4] Hankin B.L. (2006). Adolescent depression: description, causes, and interventions. Epilepsy Behav..

[5] Hankin B.L., Young J.F., Abela J.R., Smolen A., Jenness J.L., Gulley L.D., Technow J.R., Gottlieb A.B., Cohen J.R., Oppenheimer C.W. (2015). Depression from childhood into late adolescence: influence of gender, development, genetic susceptibility, and peer stress. J. Abnorm. Psychol..

[6] Pedersen H., Grønnæss I., Bendixen M., Hagen R., Kennair L.E.O. (2022). Metacognitions and brooding predict depressive symptoms in a community adolescent sample. BMC Psychiatry.

[7] Angold A., Erkanli A., Silberg J., Eaves L., Costello E.J. (2002). Depression scale scores in 8-17-year-olds: effects of age and gender. JCPP (J. Child Psychol. Psychiatry).

[8] Twenge J.M., Nolen-Hoeksema S. (2002). Age, gender, race, socioeconomic status, and birth cohort differences on the children's depression inventory: a meta-analysis. J. Abnorm. Psychol..

[9] Halbreich U., Kahn L.S. (2001). Role of estrogen in the aetiology and treatment of mood disorders. CNS Drugs.

[10] Steiner M., Dunn E., Born L. (2003). Hormones and mood: from menarche to menopause and beyond. J. Affect. Disord..

[11] Skovlund C.W., Morch L.S., Kessing L.V., Lidegaard O. (2016). Association of hormonal contraception with depression. JAMA Psychiatry.

[12] McCloskey L.R., Wisner K.L., Cattan M.K., Betcher H.K., Stika C.S., Kiley J.W. (2021). Contraception for women with psychiatric disorders. Am. J. Psychiatr..

[13] Wells A. (2009).

[14] Aubé J., Fichman L., Saltaris C., Koestner R. (2000). Gender differences in adolescent depressive symptomatology: towards an integrated social-developmental model. J. Soc. Clin. Psychol..

[15] Kockler M., Heun R. (2002). Gender differences of depressive symptoms in depressed and nondepressed elderly persons. Int. J. Geriatr. Psychiatr..

[16] Kendler K.S., Gardner C.O., Neale M.C., Prescott C.A. (2001). Genetic risk factors for major depression in men and women: similar or different heritabilities and same or partly distinct genes?. Psychol. Med..

[17] Thomas J.T., Thorp J.G., Huider F., Grimes P.Z., Wang R., Youssef P., Coleman J.R.I., Byrne E.M., Adams M., van Loo H.M., Penninx B.W.J.H., Medland S.E., Hickie I.B., Olsen C.M., Whiteman D.C., Whalley H.C., Penninx B.W.J.H., van Loo H.M., Derks E.M., The G.S. (2025). Sex-stratified genome-wide association meta-analysis of major depressive disorder. Nat. Commun..

[18] Jose P.E., Brown I. (2008). When does the gender difference in rumination begin? Gender and age differences in the use of rumination by adolescents. J. Youth Adolesc..

[19] Nolen-Hoeksema S., Parker L.E., Larson J. (1994). Ruminative coping with depressed mood following loss. J. Pers. Soc. Psychol..

[20] Birmaher B., Ryan N.D., Williamson D.E., Brent D.A., Kaufman J., Dahl R.E., Perel J., Nelson B. (1996). Childhood and adolescent depression: a review of the past 10 years. Part I. J. Am. Acad. Child Adolesc. Psychiatry.

[21] de Muinck Keizer-Schrama S.M.P.F. (2007). Introduction and management of puberty in girls. Horm. Res..

[22] Anderl C., de Wit A.E., Giltay E.J., Oldehinkel A.J., Chen F.S. (2022). Association between adolescent oral contraceptive use and future major depressive disorder: a prospective cohort study. JCPP (J. Child Psychol. Psychiatry).

[23] Berenson A.B., Odom S.D., Breitkopf C.R., Rahman M. (2008). Physiologic and psychologic symptoms associated with use of injectable contraception and 20 microg oral contraceptive pills. Am. J. Obstet. Gynecol..

[24] Keyes K.M., Cheslack-Postava K., Westhoff C., Heim C.M., Haloossim M., Walsh K., Koenen K. (2013). Association of hormonal contraceptive use with reduced levels of depressive symptoms: a national study of sexually active women in the United States. Am. J. Epidemiol..

[25] Noachtar I.A., Frokjaer V.G., Pletzer B. (2023). Mental health symptoms in oral contraceptive users during short-term hormone withdrawal. JAMA Netw. Open.

[26] Schmidt P.J., Ben Dor R., Martinez P.E., Guerrieri G.M., Harsh V.L., Thompson K., Koziol D.E., Nieman L.K., Rubinow D.R. (2015). Effects of estradiol withdrawal on mood in women with past perimenopausal depression: a randomized clinical trial. JAMA Psychiatry.

[27] Buggio L., Barbara G., Facchin F., Ghezzi L., Dridi D., Vercellini P. (2022). The influence of hormonal contraception on depression and female sexuality: a narrative review of the literature. Gynecol. Endocrinol..

[28] Chen S., Gao L., Li X., Ye Y. (2021). Allopregnanolone in mood disorders: mechanism and therapeutic development. Pharmacol. Res..

[29] Pluchino N., Luisi M., Lenzi E., Centofanti M., Begliuomini S., Freschi L., Ninni F., Genazzani A.R. (2006). Progesterone and progestins: effects on brain, allopregnanolone and β-endorphin. J. Steroid Biochem. Mol. Biol..

[30] Schüle C., Nothdurfter C., Rupprecht R. (2014). The role of allopregnanolone in depression and anxiety. Prog. Neurobiol..

[31] Bäckström T., Bixo M., Johansson M., Nyberg S., Ossewaarde L., Ragagnin G., Savic I., Strömberg J., Timby E., van Broekhoven F., van Wingen G. (2014). Allopregnanolone and mood disorders. Prog. Neurobiol..

[32] Standeven L.R., McEvoy K.O., Osborne L.M. (2020). Progesterone, reproduction, and psychiatric illness. Best Pract. Res. Clin. Obstet. Gynaecol..

[33] Schaffir J., Worly B.L., Gur T.L. (2016). Combined hormonal contraception and its effects on mood: a critical review. Eur. J. Contracept. Reprod. Health Care.

[34] Kelly S., Davies E., Fearns S., McKinnon C., Carter R., Gerlinger C., Smithers A. (2010). Effects of oral contraceptives containing ethinylestradiol with either drospirenone or levonorgestrel on various parameters associated with well-being in healthy women: a randomized, single-blind, parallel-group, multicentre study. Clin. Drug Invest..

[35] Civic D., Scholes D., Ichikawa L., LaCroix A.Z., Yoshida C.K., Ott S.M., Barlow W.E. (2000). Depressive symptoms in users and non-users of depot medroxyprogesterone acetate. Contraception.

[36] Worly B.L., Gur T.L., Schaffir J. (2018). The relationship between progestin hormonal contraception and depression: a systematic review. Contraception.

[37] Bick A.J., Louw-du Toit R., Skosana S.B., Africander D., Hapgood J.P. (2021). Pharmacokinetics, metabolism and serum concentrations of progestins used in contraception. Pharmacol. Ther..

[38] Romans S., Clarkson R., Einstein G., Petrovic M., Stewart D. (2012). Mood and the menstrual cycle: a review of prospective data studies. Gend. Med..

[39] Hill S.E. (2019). This is your brain on birth control: the surprising science of women, hormones, and the law of unintended consequences. Avery.

[40] Grøntvedt T.V., Grebe N.M., Kennair L.E.O., Gangestad S.W. (2017). Estrogenic and progestogenic effects of hormonal contraceptives in relation to sexual behavior: insights into extended sexuality. Evol. Hum. Behav..

[41] Beck A.T., Steer R.A., Carbin M.G. (1988). Psychometric properties of the beck depression inventory: twenty-five years of evaluation. Clin. Psychol. Rev..

[42] Treynor W., Gonzalez R., Nolen-Hoeksema S. (2003). Rumination reconsidered: a psychometric analysis. Cognit. Ther. Res..

[43] Papageorgiou C., Wells A. (2001). Metacognitive beliefs about rumination in recurrent major depression. Cognit. Behav. Pract..

[44] StataCorp (2023).

[45] Robakis T., Williams K.E., Nutkiewicz L., Rasgon N.L. (2019). Hormonal contraceptives and mood: review of the literature and implications for future research. Curr. Psychiatry Rep..

[46] Westhoff C.L., Heartwell S., Edwards S., Zieman M., Stuart G., Cwiak C., Davis A., Robilotto T., Cushman L., Kalmuss D. (2007). Oral contraceptive discontinuation: do side effects matter?. Am. J. Obstet. Gynecol..

